# A Protocol for a Pan-Canadian Prospective Observational Study on Active Surveillance or Surgery for Very Low Risk Papillary Thyroid Cancer

**DOI:** 10.3389/fendo.2021.686996

**Published:** 2021-06-10

**Authors:** Anna M. Sawka, Sangeet Ghai, George Tomlinson, Nancy N. Baxter, Martin Corsten, Syed Ali Imran, Eric Bissada, Rebecca Lebouef, Nathalie Audet, Maryse Brassard, Han Zhang, Michael Gupta, Anthony C. Nichols, Deric Morrison, Stephanie Johnson-Obeski, Eitan Prisman, Don Anderson, Shamir P. Chandarana, Sana Ghaznavi, Jennifer Jones, Amiram Gafni, John J. Matelski, Wei Xu, David P. Goldstein, the Canadian Thyroid Cancer Active Surveillance Study Group, Lorne Rotstein

**Affiliations:** ^1^ Division of Endocrinology, Department of Medicine, University Health Network and University of Toronto, Toronto, ON, Canada; ^2^ Joint Department of Medical Imaging, University Health Network-Mt Sinai Hospital-Women’s College Hospital, University of Toronto, Toronto, ON, Canada; ^3^ Department of Medicine, University Health Network and Institute of Health Policy, Management and Evaluation, University of Toronto, Toronto, ON, Canada; ^4^ Melbourne School of Population and Global Health, University of Melbourne, Melbourne, VIC, Australia; ^5^ Department of Otolaryngology-Head and Neck Surgery, Dalhousie University, Halifax, NS, Canada; ^6^ Division of Endocrinology, Department of Medicine, Dalhousie University, Halifax, NS, Canada; ^7^ Department of Otolaryngology-Head and Neck Surgery, l’Université de Montréal, Montreal, QC, Canada; ^8^ Division of Endocrinology, Department of Medicine, l’Université de Montréal, Montreal, QC, Canada; ^9^ Department of Otolaryngology-Head and Neck Surgery, Université Laval, Quebec City, QC, Canada; ^10^ Division of Endocrinology, Department of Medicine, Université Laval, Quebec City, QC, Canada; ^11^ Department of Otolaryngology-Head and Neck Surgery, McMaster University, Hamilton, ON, Canada; ^12^ Department of Otolaryngology-Head and Neck Surgery, Western University, London, ON, Canada; ^13^ Division of Endocrinology, Department of Medicine, Western University, London, ON, Canada; ^14^ Department of Otolaryngology-Head & Neck Surgery, University of Ottawa, Ottawa, ON, Canada; ^15^ Department of Otolaryngology-Head & Neck Surgery, University of British Columbia, Vancouver, BC, Canada; ^16^ Section of Otolaryngology-Head & Neck Surgery, Department of Surgery, University of Calgary, Calgary, AB, Canada; ^17^ Division of Endocrinology, Department of Medicine, University of Calgary, Calgary, AB, Canada; ^18^ Department of Psychosocial Oncology, University Health Network and University of Toronto, Toronto, ON, Canada; ^19^ Centre for Health Economics and Policy Analysis, McMaster University, Hamilton, ON, Canada; ^20^ Mount Sinai Hospital, University of Toronto, Toronto, Biostatistics Research Unit, University Health Network, Toronto, ON, Canada; ^21^ Dalla Lana School of Public Health, University of Toronto, Toronto, ON, Canada; ^22^ Department of Otolaryngology and Head and Neck Surgery, University Health Network and University of Toronto, Toronto, ON, Canada

**Keywords:** papillary thyroid cancer, papillary thyroid microcarcinoma, active surveillance, thyroidectomy, observational cohort study, prospective research

## Abstract

**Background:**

The traditional management of papillary thyroid cancer (PTC) is thyroidectomy (total or partial removal of the thyroid). Active surveillance (AS) may be considered as an alternative option for small, low risk PTC. AS involves close follow-up (including regularly scheduled clinical and radiological assessments), with the intention of intervening with surgery for disease progression or patient preference.

**Methods:**

This is a protocol for a prospective, observational, long-term follow-up multi-centre Canadian cohort study. Consenting eligible adults with small, low risk PTC (< 2cm in maximal diameter, confined to the thyroid, and not immediately adjacent to critical structures in the neck) are offered the choice of AS or surgery for management of PTC. Patient participants are free to choose either option (AS or surgery) and the disease management course is thus not assigned by the investigators. Surgery is provided as usual care by a surgeon in an institution of the patient’s choice. Our primary objective is to determine the rate of ‘failure’ of disease management in respective AS and surgical arms as defined by: i) AS arm – surgery for progression of PTC, and ii) surgical arm - surgery or other treatment for disease persistence or progression after completing initial treatment. Secondary outcomes include long-term thyroid oncologic and treatment outcomes, as well as patient-reported outcomes.

**Discussion:**

The results from this study will provide long-term clinical and patient reported outcome evidence regarding active surveillance or immediate surgery for management of small, low risk PTC. This will inform future clinical trials in disease management of small, low risk papillary thyroid cancer.

**Registration details:**

This prospective observational cohort study is registered on clinicaltrials.gov (NCT04624477), but it should not be considered a clinical trial as there is no assigned intervention and patients are free to choose either AS or surgery.

## Introduction

Over the last three decades, the incidence rate of thyroid cancer (TC) has been rising in Canada (1), yet the disease-related mortality rate has remained very low (1, 2). Globally, this trend has been partly explained by overdiagnosis of TC - particularly low risk disease, with an associated low risk of disease-related mortality (3). In Canada, small papillary thyroid cancers (PTCs) have been increasingly diagnosed (4). The traditional standard of care in initial PTC management has been thyroidectomy (total or partial thyroid removal by surgery) and this may be followed by thyroid hormone treatment and sometimes radioactive iodine treatment (5). However, recently the authors of the American Association of Endocrine Surgeons’ Clinical Practice Guidelines for Definitive Surgical Management of Thyroid Diseases in Adults have indicated that “an active surveillance protocol for papillary thyroid microcarcinoma may be appropriate for carefully selected, informed, and compliant patients” (6). AS has also been discussed in the American Thyroid Association (ATA) guidelines on management of differentiated thyroid cancer in adults (5). Active surveillance (AS) is defined by close clinical follow-up instead of immediate surgery, including regularly scheduled neck ultrasound imaging, with the intention for surgery (with curative intent) if there is evidence of disease progression while under observation or if the patient requests surgery for another reason. Offering the option of AS to patients with small, low risk PTC may mitigate the risk of treatment-related morbidity for disease that may ultimately never progress, yet still allow for curative treatment if early progression is detected in the surveillance process.

Recent literature suggests that AS of small, low risk PTC (confined to the thyroid), is safe (7). In a recent systematic review and meta-analysis of long-term outcomes of patients with small, low risk PTC under AS, Cho et al. reported that only 5.3% of patients experienced tumor growth ≥3 mm in maximal diameter and 1.6% of patients developed nodal metastases during 5 years of follow-up (7). No thyroid cancer-related deaths nor incident distant metastases have been observed in patients with small, low risk PTC under AS (8). However, most of the published literature on AS includes primarily patients with papillary microcarcinoma (PTC 1cm or smaller in largest diameter) (7), which may not routinely necessitate biopsy, per recent ATA clinical practice guidelines guidance (5). In 2016, in Toronto, we initiated a multi-phase prospective observational study of patients with small, low risk PTC < 2cm in maximal diameter (i.e. including PTC larger than microcarcinoma), which we refer to as very low risk PTC (VL-PTC) (9). In the Toronto study, we offer patients a choice of surgery or AS and report their disease management decision (decision-making phase of the study) (9). We also prospectively report patient outcomes in patients choosing AS or surgery (Clinicaltrials.gov: NCT03271892) (9). An interim analysis on the disease management decisions of the first 100 patients enrolled in the Toronto study was recently published (10). About half of the enrolled patients in the Toronto study have a PTC larger than a microcarcinoma (10). We reported that 71.0% (71/100) of our patients chose AS over surgery and patients’ rationale for the decision was described in detail using a qualitative approach (10). Although the high percentage selecting AS may in part reflect some referral bias (both self-referral of patients who are interested in AS and referral from thyroid cancer specialists whose patients specifically request AS), these preliminary data suggest that among Canadians with VL-PTC there is substantial interest in being offered the choice of AS as an alternative to surgery (10). One of the main reasons why our patients chose AS, was a desire to avoid immediate thyroid surgery, with the potential implications of thyroid hormone treatment (10). In contrast, patients who chose surgery wanted to remove any thyroid cancer in their body (i.e. aspired for cure of the disease). Recruitment in the Toronto prospective, observational AS decision-making study is now complete (200 patients), (with a full report on disease management decisions forthcoming). Furthermore, long-term patient follow-up is ongoing in the Toronto study. In response to a) popularity of the option of AS in patients with small, low risk PTC in the Toronto study (10), b) recently acquired knowledge in this area (in the published literature and clinical practice guidelines), and c) recent interest among Canadian thyroid cancer specialists, we have developed a prospective observational pan-Canadian study examining long-term clinical and patient-reported outcomes on AS and surgery for VL-PTC. The proposed study, described below, is similar to design to the original Toronto study, but with a focus on clinical outcomes and patient reported outcomes over a long period of follow-up.

## Materials and Methods

### Study Design and Setting

We plan a multi-centre prospective observational cohort study to examine long-term outcomes of patients with small, low risk PTC, confined to the thyroid, who have made an initial choice of AS or surgery. A draft of this study protocol was presented at a national investigator meeting held in Toronto on November 30, 2019 (sponsored by a Canadian Institutes of Health Research (CIHR) planning grant) (11) and the protocol was then refined, based on feedback from the investigators at the meeting. The nine planned participating institutions include: University Health Network/University of Toronto (Toronto, Ontario), Centre Hospitalier de l’Université de Montréal (Montreal, Quebec), Dalhousie University (Halifax, Nova Scotia), Université Laval (Quebec City, Quebec), McMaster University (Hamilton, Ontario), University of British Columbia (Vancouver, British Columbia), University of Calgary (Calgary, Alberta), University of Ottawa (Ottawa, Ontario), and Western University (London, Ontario).

### Study Aim and Primary Outcomes

Our primary research objective is to quantify the safety of AS relative to immediate surgery, by examining the rate of ‘failure’ of disease management over the period of follow-up in VL-PTC patients choosing AS and those choosing surgery. ‘Failure’ of disease management is defined as follows in each arm of the study: i) AS arm – thyroid surgery for progression of cancer, and ii) Surgical arm - surgery or other thyroid cancer treatments for structural disease recurrence or persistence at any time point after completion of initial treatment. Treatment of recurrent or persistent thyroid cancer (after completion of initial treatment) may include additional surgery, radioactive iodine treatment, ethanol ablation of lymph nodes, or external beam radiation treatment. Data will be reported separately for each arm.

### The Study Population Eligibility Criteria and Recruitment

Consenting adults (age ≥18 years) with surgically untreated PTC < 2cm in maximal diameter that is confined to the thyroid, not encroaching on critical structures in the neck (e.g. trachea or recurrent laryngeal nerve), with no evidence of extrathyroidal extension nor nodal metastases (which we refer to as very low risk PTC, VL-PTC), are eligible for inclusion in the study (**Table 1**). Several minor changes in the original Toronto inclusion and exclusion criteria are also detailed in **Table 1**, largely enabling recruitment of interested patient groups (such as those with other low risk malignancies or other thyroid or parathyroid disease for which there is no absolute indication for surgery– see **Table 1**). Data from patients who consented to long-term follow-up in the original Toronto study (NCT03271892) will be reported with data from the additional patients recruited in the multi-centre study (NCT04624477).

**Table 1:** Eligibility criteria.

Participant Inclusion and Exclusion Criteria	
*Inclusion criteria*	*Exclusion criteria*
•Age 18 years or older	•Thyroid cancer that is known to have extended beyond the thyroid (e.g. extrathyroidal extension, nodal or distant metastases)
•Newly diagnosed, untreated papillary thyroid cancer (PTC) < 2cm in maximal diameter on ultrasound imaging. The fine needle aspiration biopsy cytology of the primary tumor is required to be read as PTC or suspicious for PTC*	•A history of prior thyroid surgery*
•Absence of metastatic cervical lymphadenopathy on imaging (ultrasound the neck or other)	•The primary PTC is encroaching the recurrent laryngeal nerve or trachea (which is considered at potential high risk for invading these structures in the event of disease progression)
•No other absolute* indication for thyroid or parathyroid surgery at the time of the assessment	•Proven or suspected poorly differentiated or non-papillary thyroid cancer
•Patient must grant permission for review of thyroid cancer-related medical records	Medically unfit for surgery due to severe co-morbidity. Severe comorbidity may include another active malignancy with limited life expectancy of < 1 year*
	•Pregnancy at the time of study enrollment
	•One or more absolute indications for thyroid or parathyroid surgery (other than thyroid cancer)*
	•Patient is unable or unwilling to consent for study follow-up procedures (e.g. due to current severe active cognitive or psychiatric impairment, substance abuse, or other reasons)

*Changes in the original Toronto protocol, approved by the University Health Network Research Ethics Board (April 7, 2020) and incorporated in the pan-Canadian at onset of the study include: not mandating central cytopathology review (i.e. may be performed at the discretion of the investigator if there is any uncertainty or concern about the original review, but not mandating this due to limited value with some patient delays observed in the Toronto study), not excluding patients who have mild thyroid or parathyroid disease that does not meet established absolute indications for surgical treatment (e.g. hyperthyroidism under control with medication, mild primary hyperparathyroidism), excluding patients with any prior thyroid surgery (not just thyroid cancer surgery, as prior partial thyroidectomy could confound results at long-term follow-up, although no patients with prior thyroid surgery have been enrolled in the Toronto study), and permitting patients who have had a recent diagnosis, treatment, or active surveillance of another malignancy to participate in the study if the expected survival of the other malignancy is > 1 year (as patients with other malignancies, including those under active surveillance for other low risk cancers, have expressed interest in this study).

Patient recruitment at all study sites is focused in participating thyroid cancer clinics, although eligible patients may be referred by healthcare providers or refer themselves by contacting a study centre. Screening for eligibility in consenting patients is performed by a research assistant, under the supervision of a site investigator. All patients are required to have at least one consultation by a thyroid cancer surgeon (at any institution) such that the type of surgery that would be recommended for the particular case, as well as potential complications, are explained in detail (as per usual clinical practice of the surgeon). Participants are informed about the options of AS and surgery and provided written handout summarizing the relevant literature on both AS and surgical options, and given the opportunity to meet with a study physician to discuss the provided information and obtain answers to any related questions. Patients are free to make their own disease management choice and the clinical disease management course (i.e. surgery or AS) is not assigned by the investigators in the study. Baseline neck imaging studies are reviewed by a study investigator experienced in interpretation of neck ultrasound or a radiologist with expertise in ultrasound who is familiar with the study, to confirm eligibility. Training on evaluation of ultrasounds for baseline assessment and follow-up has been provided at a national investigator meeting (SGhai, DPG) and will be revisited during the course of the study at yearly (or more frequent) investigator meetings. The central study co-primary investigators (AMS, DPG) are available to advise and support the other investigators throughout the study. A REDCap database has been developed by the THETA Group at University Health Network, for centralized electronic collection of data entered by research staff at each study site.

### Study Follow-Up Assessments and Outcomes

As in the original study (9), AS routine follow-up includes clinical assessment (comprised of neck ultrasound at the participating institution, bloodwork (TSH, thyroglobulin, thyroglobulin antibody), and investigator clinical evaluation) every 6 months for two years, then yearly if there has been no evidence of disease progression. More frequent assessments or additional investigations may be performed if there is a clinical concern. Indefinite clinical follow-up at a participating study site is offered for patients as long as they are undergoing AS. The criteria for recommending surgery for disease progression (the same as in the original study) are defined as any of: 1) primary tumor growth > 3mm (in largest diameter, confirmed on two consecutive ultrasound exams); 2) PTC growth in a location that is concerning (i.e. encroaching the trachea or course of the recurrent laryngeal nerve or grossly invading extrathyroidal tissues); or 3) incident nodal metastatic disease, or 4) incident distant metastatic disease (**Table 2**) (9). Furthermore, as in the original Toronto study, patients under AS are free to choose to cross over to surgery in the absence of disease progression at any point in follow-up and may continue to be followed in the study (in a manner analogous to that of patients who choose immediate surgery) (9). In patients initially choosing surgery or crossing over from AS to surgery, the extent of surgery and any other treatments will be at the discretion of the treating surgeon or thyroid cancer specialists (who are not mandated to be at a participating study site), and is intended to reflect usual care. Typical post-surgical disease surveillance of patients treated for VL-PTC includes periodic clinical evaluations, neck ultrasounds, and bloodwork (TSH, thyroglobulin, thyroglobulin antibody), with additional investigations considered depending on the clinical circumstance. In the event that the clinical criteria for salvage surgery are met, it is expected that the treating or referring surgeon will perform thyroid surgery, but the patient may opt for a surgeon of her or his choice at any institution (usual care). Although highly unlikely, it is possible that a patient may decline surgery (e.g. due to development of new co-morbidity, or other reasons). Any occurrence of non-compliance with a recommendation for salvage surgery will be recorded, including the reason. However, based on our experience in Toronto, although sometimes patients may delay recommended surgery (e.g. due to life circumstances or COVID-19 pandemic related reasons), we have not had any patient completely refuse surgery and to date, ultimately all patients who were advised to have surgery by study investigators have done so. In the case of patients who initially decline surgery, close AS follow-up will continue, with imaging and clinical follow-up frequency per discretion of the study investigator (typically 3 to 6 months).

**Table 2:** Definition of disease progression.

Definition of progression of papillary thyroid cancer under active surveillance for which salvage surgery is advised (one or more of the criteria listed below)*
1. Primary index PTC growth >3 mm (in the largest dimension), confirmed on two consecutive ultrasound exams. The 3 mm size cut off is considered reproducible (21)
2. Primary PTC growth in a location that is concerning (e.g. immediately adjacent to the trachea or in the course of the recurrent laryngeal nerve) (22)
3. Incident development of metastatic PTC to lymph nodes (confirmed on cytology or unequivocal imaging)
4. Incident development of distant metastatic PTC (confirmed on imaging or biopsy or surgical histology)

*Patients may choose to have surgery in absence of disease progression at any time point in follow-up.

Thyroid cancer-related medical records will be reviewed by study staff at least yearly, for up to 10 years, regarding thyroid cancer outcomes, treatments, and potential treatment-related complications. Patient questionnaires/interviews and communication scripts are in English for most study sites, but in (Quebec/Canadian) French for Quebec sites.

For patients recruited in the multi-centre study, a baseline self-administered written questionnaire includes demographics and medical history, a question on baseline disease management preference, as well as the following patient-reported outcomes: i) quality of life (thyroid cancer/cancer-specific - EORTC QLQ-THY34 (12) with EORTC QLQ-C30 (13) and ii) anxiety (Generalized Anxiety Disorder Screener, GAD-7) (14). Patients recruited from the original Toronto study who had previously completed a baseline questionnaire as outlined in the original study protocol (10), will not perform a repeat baseline assessment, but relevant original baseline data will be reported.

Yearly follow-up questionnaires (up to 5 years) include: an update of thyroid cancer status and thyroid cancer treatments as well as the following questionnaires: i) quality of life (thyroid cancer/cancer-specific - EORTC QLQ-THY34 (12) with EORTC QLQ-THY34 (13) ii) anxiety (Generalized Anxiety Disorder Screener, GAD-7) (14), iii) survivors’ concerns (Assessment of Survivor Concerns, ASC [1])), and iv) decision regret, relating to the original decision to undergo AS or surgery [decision regret scale (15)]. The questionnaires used in this study to evaluate quality of life and psychosocial outcomes have all been previously used in cancer populations.

For sites in the province of Quebec using French language EORTC QLQ-THY-34 (12) and ASC (16) adapted to Quebec French are not available at the time of initiation of this protocol so they will be initially omitted, with the intention of incorporating them once available (after approval of research ethics board amendments in sites in the province of Quebec).

### Sample Size Calculation

Statistical assumptions justifying our proposed total sample size of 450 patients (in total, including consenting patients from the original Toronto study as well as patients from all participating sites recruited in the multi-center study) are as follows, as they relate to the primary outcome analysis: i) Based on our current preliminary data from Toronto (September, 2020), the ratio of those choosing AS to those choosing surgery will be 3:1. ii) Based on published data from a prior published retrospective study of Toronto patients under the care of a primary investigator (AMS), it is expected that within an approximately 5-year time frame, approximately 3.4% of low risk papillary thyroid cancer patients in the surgical arm will receive treatment for recurrent disease (17). For purpose of sample size calculations this number is rounded down to 3%, in the time frame of about 2 to 3 years. Based on published data on active surveillance of VL-PTC, which is largely based on papillary microcarcinomas (7), we estimate that after about 2 - 3 years of follow-up, the cumulative incidence of disease progression requiring surgery in the AS arm will be approximately 3% (including both tumor growth and incident nodal metastatic disease). The sample size is guided by estimation of disease management ‘failure’ (absence of success) in the AS arm, as outcomes with surgery are better established. To estimate the risk of ‘failure’ of disease management in the AS group with an expected 95% confidence interval half-width of 2% (e.g., a confidence interval of 2% around the estimate of 3%), the required sample size is 328; there is an 80% chance the CI half-width will be narrower than 2.1%. Under the 3:1 ratio assumption above, approximately 110 surgical patients need to be recruited to obtain 328 AS patients. In order to accommodate some attrition in both groups, we plan to recruit a total of 450 patients (including patients consenting to long-term follow-up from the original Toronto study) and expect approximately 330 in AS group and 120 in the surgical group). Recruitment of surgical arm patients will enable controlled comparisons for the secondary outcomes. The sample size will be re-evaluated on a yearly basis, and the total sample size may be adjusted accordingly to maintain the desired precision in the AS arm.

### Statistical Analysis

Descriptive data will be presented in the surgical and AS groups for baseline demographic characteristics, medical history, initial disease management choice (surgery or AS), patients’ rationale for their choice, thyroid cancer treatments, and thyroid hormone treatment. Summaries of follow-up date will tabulate major complications of thyroid cancer surgery (i.e. clinically diagnosed hypoparathyroidism or recurrent laryngeal nerve injury beyond the first year after any thyroid cancer surgery), most recent PTC disease status, proportions crossing over from AS to surgery (and indication), and deaths (thyroid cancer-specific and overall, respectively).

The primary analysis will separately estimate the cumulative incidence of ‘failure’ of disease management (along with 95% confidence intervals) at any given time since enrolment in the AS and surgical arms, using the Kaplan-Meier method. The differences in cumulative incidence will also be calculated (with 95% confidence intervals).

For categorial outcomes, we will report proportions with 95% confidence intervals according to AS or surgical group. For all categorical clinical outcomes for which there is a sufficient number of events for meaningful analysis, we will compare the AS and surgical groups using competing risks models. We will summarize the following secondary patient-reported outcomes in each group: i) quality of life (thyroid cancer/cancer-specific) (12, 13), ii) anxiety (14), iii) survivor concerns (16), and iv) decision regret (15) (relating to the original decision on VL-PTC disease management). The data will be reported at last available study follow-up, with data from prior time points analyzed in interim analyses, if there are sufficient patients and events for meaningful analysis. All questionnaire data will be scored as per developers, for total scores or subscale scores. Where patient reported outcomes are missing and patients are known to be alive, multiple imputation will be used, with other outcomes one the same patient at the same time or other times in the imputation model. For each questionnaire outcome, we will calculate summary statistics to characterize the cohort at the last follow-up. Analyses will be adjusted for baseline variables, including age, sex, and primary tumor size (≤ 1cm or > 1cm) as well as duration of follow-up. If sufficient data are available for meaningful analysis will examine predictors of psychosocial health outcomes (e.g. age, sex, primary tumor size) if sufficient data are available for meaningful analysis. Clinical outcomes will also be compared between men and women, if there are sufficient events.

### Informed Consent, Ethics Review, and Data Safety and Monitoring

The original single-centre study in Toronto was approved by the University Health Network Research Ethics Board (ID 15-8942); expanded participant recruitment in Toronto (beyond the original sample size of 200 participants in 15-8942) and the addition of other Ontario sites is provincially approved by the Ontario Cancer Research Ethics Board (ID 1986). At the time of writing of this manuscript, the Ontario Research Ethics Board has approved the Toronto, Ottawa, and London, Ontario sites, with the Hamilton application awaiting completion by the site investigator. Respective research ethics board approval has also been obtained at the following sites: Halifax, Quebec City, and Vancouver. Otherwise, individual research ethics board applications are in progress for other planned participating sites (Hamilton, Calgary, and Montreal).

Aggregate data on the study recruitment rate in both arms, rates of surgery, AS, and AS cross-over, and thyroid cancer outcomes as well as serious adverse events (defined by death due to thyroid cancer or development of distant metastatic thyroid cancer) will be reported yearly to an independent Data and Safety Monitoring Committee (DSMC) made up of representatives who are independent of investigators. The DSMC will provide a yearly summary, outlining any concerns or suggestions, and providing approval for continuation of the study.

## Discussion

Management of small, low risk PTC is evolving, with a tendency to less aggressive treatment in recent years, consistent with recommendations of the 2015 American Thyroid Association guidelines (5). In 2014, we surveyed Canadian thyroid cancer specialists about their recommendations in the initial management of low risk papillary microcarcinoma, and only 2% of respondents endorsed active surveillance (18). The three most important reasons that Canadian thyroid cancer physicians reported in explaining their recommendations were: 1) consideration of patient clinical risk factors, 2) the ability to cure the patient surgically, and 3) the perception of low morbidity in association with thyroid surgery (18). Physicians rated as less important, consideration of patient’s comfort level with active surveillance of thyroid cancer and patient’s disease management preference (18). Yet at the time of this physician survey, there was relatively little knowledge as to whether Canadian low risk PTC patients’ preferences regarding initial disease management. We thus initiated the Toronto study, determining how often Canadians with small, low risk PTC would choose AS, if offered as a research alternative to surgery (and why) (funded by the Ontario Ministry of Health) as well as their level of decisional regret 1 year after being presented with the choice (funded by the Canadian Cancer Society) (9). First, in the Greater Toronto Area and then nationally, we initiated discussions among thyroid cancer specialist physicians and surgeons on providing the option of AS for small, low risk PTC, in a clinical research context. The Greater Toronto Area Canadian Thyroid Cancer Active Surveillance Study Group Investigators thus established a research consortium, to facilitate patient participant recruitment and input in the study. Input and participant recruitment support from this group has been instrumental in the success of the initial study and informed the design of the subsequent multi-centre study described in this manuscript. A national investigator meeting on this topic was held in Toronto in November of 2019, involving potential site primary investigators from other Canadian cities (11). Importantly, patient stakeholders shared their views on initial management of small, low risk PTC, their personal experiences, and their shared guidance in continuing to enable patients to be involved in medical decision-making in this regard (11). Furthermore, our early observations in the Toronto study on patients’ rationale for AS or surgery has also informed us as to what outcomes are important to patients and informed our understanding that the goals of patients choosing AS are very different from those choosing immediate surgery (10). We thus learned that, although one of the reasons rated by Canadian thyroid cancer physicians as being most important in disease management of small, papillary thyroid microcarcinoma was the ability to cure the malignancy (18), we later found that this was not necessarily a priority for all patients (10).

Low risk PTC patients’ rationale for AS or surgery is important to understand. Our preliminary findings on patients’ rationale for AS (10) confirmed prior observations reported by D’Agostino et al. at Memorial Sloan Kettering, who reported that some patients perceived the cancer as relatively indolent and experienced concerns related to living without a thyroid or requiring thyroid hormone treatment (19). Concerns about taking thyroid hormone are commonly expressed by patients considering thyroid surgery. A recent international survey of more than 12,000 patients on thyroid hormone treatment suggested prominent dissatisfaction in about 15% of patients taking levothyroxine (20). Although only a small subset of surveyed patients had a history of thyroid cancer (346 patients), thyroid cancer patients reported a high level of agreement with a statement indicating that thyroid hormone treatment impacted their lives (20). The concerns that are expressed by some thyroid cancer patients regarding the long-term implications of thyroidectomy and thyroid hormone treatment are important for healthcare practitioners to acknowledge and formally address in the discussion of disease management choices. However, it is equally important to acknowledge that for some patients, their primary goal in managing their disease is cure of the cancer (by surgery and other treatments, if needed) (10). For informed, low risk PTC patients who are not comfortable living with the knowledge of an untreated thyroid cancer, surgery is clearly the preferred initial disease management choice (10). Appropriate disease management of small, low risk PTC should thus incorporate consideration of patients’ preferences.

Some strengths of our proposed research include prospective design, detailed prospective reporting of patients’ disease management choice, the use of a well-established AS protocol, evaluation of patient reported outcomes, collection of long-term follow-up data, and a relatively large sample size (combining data from the original Toronto study and multi-centre study). An important limitation is that the study is not a randomized controlled trial. Yet by enabling patients to select their disease management choice, we will be able to examine if the study outcomes align with the long-term goals of the patients. We anticipate that our research will inform the evidence basis for counseling patients with small, low risk PTC on the long-term outcomes of AS or surgery and advance patient-centered care in thyroid oncology.

## Ethics Statement

The original single-centre study in Toronto was approved by the University Health Network Research Ethics Board (ID 15-8942); expanded participant recruitment in Toronto (beyond the original sample size of 200 participants in 15-8942) and the addition of other Ontario sites is provincially approved by the Ontario Cancer Research Ethics Board (ID 1986). At the time of writing of this manuscript, the Ontario Research Ethics Board has approved the Toronto, Ottawa, and London, Ontario sites, with the Hamilton application awaiting completion by the site investigator. Respective research ethics board approval has also been obtained at the following sites: Halifax, Quebec City, and Vancouver. Otherwise, individual research ethics board applications are in progress for other planned participating sites (Hamilton, Calgary, and Montreal). The patients/participants provide their written informed consent to participate in this study.

## Canadian Thyroid Cancer Active Surveillance Study Group Investigators (Greater Toronto Area Investigators): University Health Network

Lorne Rotstein, Dale Brown, John de Almeida, Patrick Gullane, Ralph Gilbert, Douglas Chepeha, Jonathan Irish, Jesse Pasternak, Shereen Ezzat, James P. Brierley, Richard W. Tsang; **Mount Sinai Hospital ,** Eric Monteiro, Afshan Zahedi, Jacqueline James, Karen Gomez Hernandez; **Sunnybrook Health Sciences Centre**, Antoine Eskander, Danny Enepekides, Kevin Higgins, Ilana J. Halperin; **Women’s College Hospital**, Afshan Zahedi, Karen Devon; **North York General Hospital**, Everton Gooden, Manish Shah; **William Osler Health System**, Mark Korman; **Trillium Health Partners**, Janet Chung, Kareem Nazarali; **Lakeridge Health**, Eric Arruda, Artur Gevorgyan; **Rouge Valley (Centenary Site)**, Michael Chang; **Scarborough Hospital (Grace Site)**, Sumeet Anand; **Scarborough Hospital (General Site)**, Vinay Fernandes; **Guelph General Hospital**, Denny Lin; **Grand River Hospital (Kitchener)**, Avik Banerjee, Vinita Bindlish, Vinod Bharadwaj, Maky Hafidh; **Humber Hospital**, Raewyn Seaburg, Laura Whiteacre

## Author Contributions

AMS and DPG (co-primary investigators) designed the study, secured research funding, and oversee all aspects of execution and reporting of the Toronto and multi-centre study. SGhai is the primary study radiologist, involved in study design and oversight of radiologic procedures and outcomes. All of the site (co-)primary investigators/collaborators have provided input in study design, will be active in assisting in participant recruitment for the study (after obtaining institutional research ethics board approval), and have provided input on this manuscript (MC, SI, EB, RL, NA, MB, HZ, MG, AN, DM, SJ-O, EP, DA, SC, SGhai, SGhaz). NB, GT, JM, JJ, WX, and AG have provided input in methodologic aspects of the study design and analysis/interpretation of results. JM, GT, and WX are statisticians who provided input on the sample size calculation and the analysis of the study results. All authors contributed to the article and approved the submitted version. The Canadian Thyroid Cancer Active Surveillance Study Group Investigators (Greater Toronto Area Investigators) have been active in participant recruitment in the original Toronto study and will be active in recruitment for the Toronto site as part of the multi-centre study.

## Funding

The original, single-centre Toronto study was funded by an operating grant (Innovation Grant) from the Ontario Academic Health Sciences Centres Alternate Funding Plan Innovation Fund (Ontario Ministry of Health) and a Lotte and John Hecht Memorial Foundation Innovation Grant from the Canadian Cancer Society (#703948). A national investigator meeting for the pan-Canadian study was funded by a Planning and Dissemination Grant from the Canadian Institutes of Health Research (FRN PCS 161831) and the Princess Margaret Cancer Centre Endocrine Oncology site group. The multi-centre study is funded by a Project Grant from the Canadian Institutes of Health Research (PJT-162314) and an Innovation to Impact Grant from the Canadian Cancer Society (706302).

## Conflict of Interest

The authors declare that the research was conducted in the absence of any commercial or financial relationships that could be construed as a potential conflict of interest.
